# The evolution of the Institute of Evolutionary Medicine at the University of Zurich (10-year anniversary)

**DOI:** 10.1093/emph/eoag003

**Published:** 2026-01-20

**Authors:** Abigail E Colby, Shania Lüthold, Nicole Bender, Frank Rühli

**Affiliations:** Institute of Evolutionary Medicine, University of Zurich, Zurich, Switzerland; Institute of Evolutionary Medicine, University of Zurich, Zurich, Switzerland; Institute of Evolutionary Medicine, University of Zurich, Zurich, Switzerland; Institute of Evolutionary Medicine, University of Zurich, Zurich, Switzerland

**Keywords:** institute of evolutionary medicine, anniversary, Zurich, Switzerland

## INTRODUCTION

**Figure 1:**
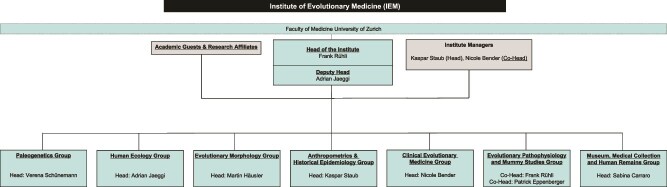
The organigram of the IEM showing the seven research groups with their main topics and heads.

The Institute of Evolutionary Medicine (IEM) celebrated its 10th anniversary on September 20, 2024, at the University of Zurich (UZH). Themed ‘Past, Present, Future,’ the event reflected a decade of pioneering research and interdisciplinary collaboration at the nexus of evolutionary biology and medicine. Today the IEM is an internationally leading academic center for research and teaching in evolutionary medicine, embedded in the Medical Faculty of UZH, Switzerland, and covers a vast field of research. At the IEM, researchers investigate diverse topics including paleopathology, ancient DNA of human pathogens, human ecology in non-industrial societies, evolution of bipedalism and human birth, historical development of human health and past pandemics, and human health in the light of modern mismatches. Furthermore, the IEM hosts one of the largest medical collections in Europe, and a significant collection of historical human remains (see Fig. 1 and the website (https://www.iem.uzh.ch/en.html) for a description of the research groups and topics, as well as publications, e.g. [1–10]). Frank Rühli launched the ‘Swiss Mummy Project’ in the 1990s, demonstrating the value of studying mummified remains to understand historical diseases. This project eventually led to the creation of the Center of Evolutionary Medicine in 2010, as part of the Anatomical Institute of UZH. In 2014, it gained independence and became the IEM with Frank Rühli as full professor and chair holder, and establishing the institute as a global hub for research, teaching, and service.

The IEM organizes several undergraduate courses on evolutionary medicine at the Medical Faculty as well as at the Science Faculty of UZH. Since fall 2025 evolutionary medicine is taught as part of the mandatory core curriculum in human medicine. In 2025 and in 2019, the IEM hosted two international conferences on evolutionary medicine, and it regularly organizes local European meetings, such as the interest group of German speaking evolutionary medicine researchers. A biweekly seminar series invites specialists from Switzerland and worldwide to exchange knowledge and facilitate networking and collaborations.

The anniversary in September 2024 gathered 50 participants, including senior and junior researchers, alumni, and academic guests, showcasing IEM’s interdisciplinary approach through presentations of past and ongoing projects.

## CONTRIBUTIONS FROM IEM ALUMNI

IEM alumni have played a pivotal role in the institute’s growth. Their work laid the foundation for IEM’s prominence, and many continue their careers in leading institutions worldwide, serving as ambassadors for its achievements.

Thomas Böni, former IEM co-director, gave an overview on the historical context of paleopathology in Switzerland. Swiss contributions to paleopathology date back to Felix Platter in the 16th century, who conducted the first documented analysis of ancient bones. Notable advancements followed in the 19th and 20th centuries, including Jakob Nüesch’s work on Ice Age remains, Emil Bächler’s studies on Paleolithic bones, and Otto Schlaginhaufen’s pathological findings. A major milestone was Ernst Galler’s collection, which comprises 1700 skeletal specimens showcasing rare bone diseases. This collection, now housed at IEM, continues to be an invaluable resource for understanding historical health and disease.

Christina Warinner, former research group leader at the IEM and now professor at Harvard University, discussed her research on the human oral microbiome and what it reveals about human health and history. The discovery of blue minerals in medieval dental plaque, for instance, provided evidence of women’s involvement in manuscript production. Ancient microbiome studies have also illuminated dietary shifts, such as the transition to dairy and starch consumption. While connections between microbiome evolution and human adaptations remain complex, expanding the scope of bacterial diversity research is essential to unlocking its full significance.

Christina Papageorgopoulou, former senior researcher at the IEM and now professor at Democritus University of Thrace, showed data on the population’s resilience and adaptation in preindustrial urban settings. Her work explores biocultural adaptations in historical urban populations, particularly in Thessaloniki. Spanning nearly 2000 years, the city’s history—from its origins as a polis to its evolution as a multi-ethnic metropolis—provides a unique opportunity to examine how populations adapted to urban challenges like overcrowding, disease, and social upheavals. By integrating skeletal analysis with historical documentation, researchers uncover how events such as plagues and conquests influenced stature, life expectancy, and trauma, offering insights into resilience and adaptation in urban environments.

## CONTRIBUTIONS FROM ACADEMIC GUESTS

Academic guests have enriched IEM’s research by offering unique perspectives on evolutionary influences in human biology and medicine.

Robert Martin (Field Museum Chicago) reported on his research on evolutionary biology and medicine. He emphasized that evolutionary medicine explores how understanding human evolution enhances health science. In fact, evolutionary medicine links many diseases to a mismatch between ancient adaptations and modern life. The field uses evolutionary tools like DNA analysis to track diseases, as seen with COVID-19. It also draws insights from other species, especially in reproductive biology, with examples like menstruation, maternal immunity, childbirth, and aging.

Maciej Henneberg (University of Adelaide, Australia), challenged simplistic explanations in evolutionary medicine. He critiqued the overreliance on the principle of Ockham’s Razor in evolutionary medicine, advocating for systems theory to address the complexity of living organisms. Unlike reductionist approaches, systems theory offers a holistic view, better capturing the intricate interplay of biological processes and ensuring sustainable solutions.

Luciano Saso (La Sapienza University of Rome, Italy) gave a summary on the evolution of medical education, including teaching in evolutionary medicine. He addressed the challenges posed by rapid advancements in medical knowledge. Beyond technical skills, modern healthcare demands empathy, resilience, and critical thinking. Technological innovations, such as simulation, augmented reality, and generative AI, present both opportunities and challenges in adapting learning methods and maintaining academic integrity. Balancing foundational knowledge, practical skills, and emerging technologies is essential to preparing healthcare professionals for an evolving field.

## CONTRIBUTIONS FROM IEM JUNIOR RESEARCHERS

Junior researchers are the backbone of the IEM, driving its progress with their contributions to current projects, and the institute’s future by fostering innovation.

Dominik Jud and Valerie Baettig, both Master students in the Human Ecology Group, reported on their field research in Bolivia. Research with subsistence populations is critical to evolutionary medicine as these communities often provide insights into lifestyles more representative of human evolutionary history compared to WEIRD (Western, Educated, Industrialized, Rich, Democratic) populations. One such group is the Tsimane, a horticulturalist community in the Bolivian Amazon collaborating with IEM’s Human Ecology Group. Fieldwork among the Tsimane involves challenging conditions such as extreme heat, mosquitoes, and remote locations accessible only after days of travel. This project offers unique insights into evolutionary mismatches, and how lifestyle transitions affect health and behavior across contrasting environments.

Camila Scaff, a postdoctoral scholar in the Human Ecology Group, gave an overview of evolutionary medicine insights on neurodiverse traits. Neurodiversity spans a spectrum, ranging from neurotypical to neurodiverse traits, with the latter often stigmatized due to deviations from societal norms. Research suggests that neurodiversity arises from genetic, environmental, and cultural factors. However, the majority of studies have focused on WEIRD populations, overlooking global diversity. Understanding how neurodiverse traits manifest in non-WEIRD communities, such as the Tsimane, is crucial. However, current measurement tools are tailored to WEIRD populations, limiting their cross-cultural applicability. By creating culturally valid tools, researchers aim to explore neurodiverse traits’ evolutionary origins, their relationships to biological fitness, and their historical persistence.

Asya Makhro, a postdoctoral scholar in the Paleopathology and Mummy Studies Group, as well as in the Clinical Evolutionary Medicine Group, reported on evolutionary adaptations to oxidative stress and the role of the nuclear transcription factor NRF2. NRF2 plays a pivotal role in combating oxidative damage by activating antioxidant enzymes and improving mitochondrial efficiency. While human adaptations to NRF2 remain limited, other species exhibit remarkable NRF2-related genetic changes. Arctic ground squirrels rely among others on NRF2 to endure hibernation, and herbivores utilize it to better metabolize plant toxins. Humans lack such specialized adaptations, raising questions about alternative strategies our ancestors developed to manage oxidative stress. Investigating these pathways could illuminate evolutionary mechanisms shaping human health and longevity.

Mathilde Le Vu, a PhD student in the Anthropometrics and Historical Epidemiology Group, summarized the impact of maternal infections on fetal development. Maternal infections, such as syphilis and influenza, significantly affect fetal development, challenging the traditional brain-sparing hypothesis that suggests the brain is prioritized during resource constraints. Le Vu demonstrated that syphilis exposure reduced both birth weight and brain volume more profoundly than influenza, largely due to preterm births. Influenza, by contrast, equally impacts birth weight and brain size through intrauterine growth restriction.

Sophie Ledebur and Theresa Bayer, both postdoctoral scholars in the Collections Group, gave an overview on the history of the IEM medical collection. Founded in 1915 by Dr Gustav Adolf Wehrli, the UZH medical collection became part of the IEM in 2016. Initially a teaching resource, it evolved into a cultural repository of therapeutic practices. Comprising over 35 000 objects, it is one of Europe’s most extensive medical collections, supporting research, education, and public engagement. Efforts to digitize the collection aim to preserve these treasures and enhance accessibility for future scientific endeavors.

Megan Malherbe, a PhD student in the Evolutionary Morphology Group, introduced the human remains collection at the IEM, including the Galler Collection of diseased bones, the medieval Dalheim Collection, and a wet specimen archive documenting forensic and pathological cases. Additionally, the histological collection holds samples from ancient mummies and extinct species. Despite these resources, only a fraction of Swiss museum specimens have been digitized so far. The IEM is actively working to bridge this gap, ensuring these collections support science and education.

## FUTURE OF THE IEM

At the end of the celebration, a discussion panel comprising IEM members and UZH clinicians dove into possible future developments of the IEM. They discussed that to maximize its impact, the IEM shall aim to bridge the gap between evolutionary research and clinical practice, reinforcing its collaborations with the University Hospital. For instance, research on cesarean births has revealed potential long-term consequences that are rarely considered in clinical decisions. Likewise, integrating evolutionary frameworks has improved strategies for managing antibiotic resistance and cancer. Embedding evolutionary principles in medical education and raising public awareness are essential to realizing the full potential of this discipline. Furthermore, collaborative global efforts, combined with advanced technologies like AI, promise to propel evolutionary medicine forward, at IEM and worldwide.

## CONCLUSION

The IEM’s 10th anniversary highlighted its contributions to evolutionary medicine, emphasizing its interdisciplinary approach to addressing health challenges. From its foundational research on mummies to insights into Swiss historical data and field studies in Bolivia, the IEM continues to bridge historical and contemporary medical knowledge. The IEM’s first decade reflects its vision, ‘learning from the past for the present and the future.’ By continuing its efforts in research collaborations, innovation, and education, the IEM is well-positioned to contribute to the field’s future.
